# SARS-CoV-2 Inflammatory Syndrome. Clinical Features and Rationale for Immunological Treatment

**DOI:** 10.3390/ijms21093377

**Published:** 2020-05-10

**Authors:** Marcella Prete, Elvira Favoino, Giacomo Catacchio, Vito Racanelli, Federico Perosa

**Affiliations:** 1 Systemic Rheumatic and Autoimmune Diseases Unit, Department of Biomedical Science and Human Oncology (DIMO), University of Bari Medical School, Piazza G. Cesare 11, I-70124 Bari, Italy; marcella.prete@uniba.it (M.P.); elvira.favoino@uniba.it (E.F.); giaco.catacchio@gmail.com (G.C.); 2 Unit of Internal Medicine, Department of Biomedical Sciences and Human Oncology (DIMO), University of Bari Medical School, Piazza G. Cesare 11, I-70124 Bari, Italy; vito.racanelli1@uniba.it

**Keywords:** anti-cytokine antibodies, human immunoglobulin for intravenous use, COVID-19, SARS-CoV-2, inflammation, acute respiratory syndrome, immune system targeting in COVID-19

## Abstract

The current pandemic coronavirus, SARS-CoV-2, is a global health emergency because of its highly contagious nature, the great number of patients requiring intensive care therapy, and the high fatality rate. In the absence of specific antiviral drugs, passive prophylaxis, or a vaccine, the treatment aim in these patients is to prevent the potent virus-induced inflammatory stimuli from leading to the acute respiratory distress syndrome (ARDS), which has a severe prognosis. Here, the mechanism of action and the rationale for employing immunological strategies, which range from traditional chemically synthesized drugs, anti-cytokine antibodies, human immunoglobulin for intravenous use, to vaccines, are reviewed.

## 1. Introduction and Epidemiological Data 

In December 2019, the Chinese Government officially announced a severe form of pneumonia caused by a new coronavirus. It started in Wuhan, in the province of Hubei, and spread rapidly throughout China and then all over the world. 

The World Health Organization (WHO) named the syndrome CoronaVirus Disease-2019 (COVID-19), but it was later renamed “severe acute respiratory syndrome” (SARS) Coronavirus (CoV)-2-related (SARS-CoV-2) by the coronavirus Study Group of the International Committee on Virus Taxonomy [1,2]. SARS-CoV-2 is one of the seven beta coronaviruses belonging to the coronavirus family [3,4], which are common in humans and other mammals [5].

The WHO General Director, Tedros Adhanom Ghebreyesus, declared this infection pandemic in the press conference on 11 March 2020 (at www.who.int/emergencies). 

Although most human coronavirus infections are mild, before the current COVID-19 two severe coronavirus outbreaks affected humans in the past two decades: (1) the severe acute respiratory syndrome (SARS) that was caused by the SARS-CoV virus in 2002 [6,7,8] and (2) the Middle East respiratory syndrome (MERS) that was caused by MERS-CoV in 2012 [9,10], being responsible for more than 10,000 cumulative infected cases with 10% and 37% mortality rates, respectively (www.who.int/csr/sars and www.who.int/emergencies/mers-cov). The SARS-CoV and SARS-CoV-2 strains use the same region, referred to as spike, to bind the same receptor, namely the angiotensin converting enzyme-2 [11,12]. Their spike regions differ in terms of only few amino acids [13,14]. 

Since its outbreak, the SARS-CoV-2 virus infection has spread rampantly, infecting 2,029,930 confirmed cases worldwide to date, and causing 136,320 deaths, in more than 200 countries (https://gisanddata.maps.arcgis.com, 1 April 2020).

At the time we write, the USA situation dominates the world scenario, with 639,644 clinically and laboratory confirmed cases and 30,985 deaths, followed by Spain (180,659 cases) and Italy, with 165,155 confirmed cases and the highest number of deaths, now 21,6454, then France, Germany, the United Kingdom, and China, with a prevalence rate between 0.2–0.3%. In Europe, of 978,632 confirmed cases, 84,628 have died (8.6% case fatality rate and 1,6 mortality rate) (https://gisanddata.maps.arcgis.com/, 16 April 2020).

A report on 30 March 2020, related to the 10,026 Italians who had died of coronavirus infection (https://www.epicentro.iss.it/coronavirus/), described a median age of 78 (range 30–100, InterQuartile Range—IQR 73-85; 30.8% females, median age 82). The median age was 15 years higher than that of the general SARS-CoV-2-positive population (median age 63 years). Of these 10,026 patients, 74% were aged between 74 and 89 years. Only 112 (1.1%) were younger than 50 years old and 23 patients were under 40. The latter included 15 patients with serious co-existing pathologies, six with no other comorbidities, while no clinical records were available for the remaining two patients. 

In a subgroup of 909 (of the 10,026) deceased patients, for whom complete clinical records were available, 51.7% had more than three diseases, including arterial hypertension (73.5%), diabetes mellitus (31.5%), ischemic heart disease (27.4%), chronic renal failure (23.8%), atrial fibrillation (23%), active cancer in the last five years (16.5%), and heart failure (16.4%). In this group, death was caused by the acute respiratory distress syndrome (ARDS) (96.5% of cases) that was associated to acute renal failure (25.7%), acute cardiac injury (11.6%), and/or superinfections (11.2%) (www.epicentro.iss.it/coronavirus).

## 2. Clinical Features

Clinical presentations of COVID-19 range from asymptomatic (81.4%), through mildly symptomatic with or without seasonal flu-like symptoms, to severe pneumonia (13.9%) [15]. Usually, respiratory problems manifest about one week after virus entry and dyspnea ranges from effort dyspnea to dyspnea occurring at rest [16,17]. Patients with dyspnea can revert to an asymptomatic phase or progress to ARDS, requiring positive pressure oxygen therapy and intensive care therapy [18] in 17–19.6% of symptomatic patients [19,20]. ARDS, in turn, can progress to multi-organ failure [21] and, in this phase, disseminated intravascular coagulation (DIC) can also be observed [22]. The main cause of death worldwide in infected patients is a combination of both ARDS and DIC in 13.9% of cases [23].

The ARDS-stage is preceded by a marked rise of inflammatory parameters, such as serum ferritin, C-reactive protein (CRP) levels, d-dimers, and the erythrocyte sedimentation rate, and it is characterized by severe edema of the alveolar wall and lung interstices, responsible for the ground glass picture seen at chest high resolution CT scan. When DIC occurs, d-dimers levels further increase, while increased liver and skeletal muscle enzymes and/or serum urea and creatinine indicate ongoing multiorgan failure.

Clinical recovery is possible at any of the above-mentioned stages, and it is generally associated to a complete clearance of the virus, rather than to its persistence. In the latter, rarer condition, according to preliminary studies [24], the virus can be detected for a period of up to one month. Lastly, clinical recovery from the ARDS stage is rarely achieved (2.9%) [16].

Thus, at a certain stage of the infection, in some individuals the virus becomes a powerful stimulator of inflammation at alveolar levels, leading to an alveolar capillary leak-like syndrome (CLLS), with edema, and a marked impairment of gas exchange requiring assisted ventilation.

## 3. Pathology and Laboratory Evidence of CLLS and Inflammation

Autopsy studies of the lungs of patients in advanced stages of the disease are not yet available. However, histology revealed a pattern of alveolar wall edema, proteinaceous exudates, and focal reactive hyperplasia of pneumocytes with vascular congestion, patchy inflammatory cellular infiltration, and multinucleated giant cells in two patients who underwent lung lobectomy for adenocarcinoma and subsequently tested positive for SARS-CoV-2 [25]. Post-mortem biopsies of a fifty-year-old Chinese patient who died of SARS-CoV-2 with severe acute respiratory syndrome showed evident desquamation of pneumocytes, bilateral pulmonary edema with hyaline membrane formation, interstitial mononuclear inflammatory infiltration, and multinucleated syncytial cells with atypical pneumocytes [26]. These histological findings resemble those found at histological post-mortem examination in patients during the 2002 SARS infection [27], as regards diffuse alveolar wall and airspace edema, and the presence of multinucleated cells. They suggest a common mechanism(s) underlying the clinical picture of SARS [27] and COVID-19 [25], culminating in ARDS, which is likely mediated by massive cytokines release.

Indeed, high levels of several pro-inflammatory cytokines, including IL-6, IL-1, TNF-α, have been demonstrated in advanced stage patients [18,28], supporting the hypothesis that the onset of ARDS is driven by pro-inflammatory cytokines, which are responsible for the histological changes and clinically full-blown ARDS. Among pro-inflammatory cytokines, IL-6 appears to be heavily involved, as indicated by the constantly elevated levels of CRP detected.

The detection of the aforementioned DIC supports the conclusion that the resulting disease is a potent SARS-CoV-2-induced inflammation, which can be associated to alveolar CLLS. Thus, it resembles the inflammatory syndromes that may complicate anti-phospholipid antibody syndrome (APS), namely the catastrophic anti-phospholipid syndrome (CAPs), characterized by disseminated intravascular microthrombosis [29]; or, the systemic CLLS characterized by altered capillary wall permeability [30]. Besides CAPs and CLLS, the SARS-CoV-2 inflammation, leading to ARDS, shares pathogenic mechanisms and clinical-radiological aspects with other auto-immune/-inflammatory diseases, such as juvenile idiopathic arthritis and its adult form [31,32,33] and Kawasaki disease [34]. Systemic CLLS has also been observed in Kawasaki disease [35].

These considerations have prompted clinicians to resort to off-label use of pro-inflammatory cytokine-targeted reagents to treat SARS-CoV-2-infected patients with ongoing ARDS.

## 4. Immunological Rationale for Targeting the Immune System to Fight SARS-CoV-2

Besides antipyretics, oxygen, antibiotics, and a number of SARS-CoV-2 non–specific antiviral drugs, mostly protease inhibitors (i.e., Lopinavir-Ritonavir, Remdesivir, Favipiravir), which are all still under clinical trial, only one drug with immunomodulatory properties, namely hydroxy-chloroquine, is currently being used in SARS-CoV-2 infected patients in the pre-ARDS phase, following an open-label non-randomized clinical trial [36]. In fact, the drug can inhibit natural and adaptive immunity through different, albeit not well-defined, mechanisms [37]. In addition, hydroxy-chloroquine can reduce the expression of the phosphatidyl-inositol-binding clathrin assembly protein, essential for SARS-CoV-2 uptake [37,38]. Finally, preliminary studies showed that the drug accelerates body virus clearance, due to its ability to alter intracellular pH at lysosomal levels, hence reducing the intracellular viral replication rate [39,40].

All of the remaining therapeutic approaches are aimed at preventing and/or reversing the dramatic inflammatory syndrome triggered by the virus, which ultimately leads to ARDS. 

In some case series, anti-IL6 therapy was successful in stabilizing the alveolar capillary membrane, reducing alveolar wall edema, and preventing/reversing ARDS [41,42,43,44], shortening the intensive care unit stay [45,46]. The currently available reagents that can neutralize IL-6 activity are the humanized monoclonal antibody (mAb) Tocilizumab, and the fully human mAb Sarilumab. Tocilizumab is the only one being tested so far. Even so, definitive proof of the efficacy of this therapy will only be obtained from the results of currently ongoing controlled clinical trials. These, with their main operative lines, are reported, as follows: 1. Tocilizumab mAb Vs continuous renal replacement therapy (CRRT) in the management of cytokine release syndrome in COVID-19 (TACOS) (NCT04306705) (last update posted: 17 March 2020; last access date: 9 May); 2. Tocilizumab in COVID-19 Pneumonia (TOCIVID-19), (NCT04317092) (last update posted: 7 April 2020; last access date: 9 May); 3. Favipiravir (an inhibitor of virus RNA polymerase) combined with Tocilizumab in the treatment of corona virus disease 2019 (NCT04310228) (last update posted: 10 April 2020; last access date: 9 May); and, 4. Tocilizumab in the treatment of patients with COVID-19 pneumonia (TOCIVID-19) (EudraCT Number: 2020-001110-38).

However, it cannot be excluded that small molecules, given per os, like the Janus kinases (JAK) inhibitor Baricitinib, may eventually be used instead of mAbs for two reasons: (1) the drug, at a dosage of 4 mg/day, is a selective down-modulator of JAK-1 and JAK-2 and, consequently, of the expression of the JAK down-stream proteins, called “signal transducer and activation of transcription” (STATs), both of which protein families are crucial for the IL-6-driven intracellular signal. (2) Baricitinib has shown affinity for the adaptor protein (AP2)-associated protein kinase 1 (AAK1) (AP2-AAK1), and cyclin G-associated kinase, both regulators of endocytosis and, hence, of the virus uptake [47]. Thus, the ability to block IL-6 triggered intracellular signal and virus uptake could indicate Baricitinib as a suitable drug for treating the infection.

Even so, concerns have been raised regarding the mechanism of action of this class of drugs. Indeed, JAK-1 and JAK-2 are also involved in type I and type II-IFN intracellular signals, which play a pivotal role in host defense against viruses, including SARS-CoV-2 [48]. Consequently, the inhibition of JAK-1 and JAK-2 would also decrease the host defense against the virus, as a side effect, facilitating virus spread in the host. Moreover, the optimal timing for the use of JAK-1 kinase inhibitors is a matter of speculation. It is not clear whether Baricitinib should be given in the early stage of virus infection, together with hydroxy-chloroquine and/or anti-viral drugs, or at the stage of onset of ARDS [49,50].

Ongoing clinical trials will address these issues, as well as the efficacy of Baricitinib alone or in combination with other drugs (1. Baricitinib in symptomatic Patients Infected by COVID-19: an Open-label, Pilot Study, https://clinicaltrials.gov/NCT04320277 (last update posted: 22 April 2020; last access date: 9 May); and, 2. Treatment of moderate to severe COVID-19 in hospitalized patient with Lopinavir/Ritonavir, hydroxychloroquine sulfate, Baricitinib, and Sarilumab, NCT04321993) (last update posted: 24 April 2020; last access date: 9 May).

As in juvenile idiopathic arthritis, an autoinflammatory-mediated disease, IL-1β also seems to play a strategic role in SARS-CoV-2 infection, by activating the NLRP3 inflammasome, as documented by the increased IL-1β levels in lymphocytes and in the sera of infected patients [18,51]. In a mouse model of MERS-CoV infection, expressing the human dipeptidyl peptidase 4 (hDPP4) receptor, MERS-CoV caused pyroptosis and increased IL-1β expression in macrophages [52]. IL-1 blockers, such as Anakinra (and possibly mAb Kanakinumab), may be an additional weapon against the respiratory distress syndrome in COVID-19, in view of its effectiveness in patients with severe forms of sepsis and with an inflammatory profile resembling the “macrophage activation syndrome” (MAS) [53,54]. An open label, controlled, multicenter study is analyzing the therapeutic efficacy of Anakinra, together with an anti-IFN-γ mAb Emapalumab, in order to reduce the hyper-inflammation that is caused by SARS-CoV-2 Infection (Efficacy and Safety of anti-IFN-γ and Anakinra (antiIL-1) in Reducing Hyperinflammation and Respiratory Distress in Patients With COVID-19 Infection (NCT04324021) (last update posted: 9 April 2020; last access date: 9 May).

The potential utility of therapy with human immunoglobulin for intravenous use (IVIG) has been recently reported by our group [55], based on the similar etiology and inflammatory pathogenesis of SARS-CoV-2 infection to diseases for which the use of IVIG has already been approved by the FDA or EMA (Table 1) [56,57,58,59,60,61,62,63], or for which IVIG are employed off-label (Table 2) [30,64,65,66,67,68,69,70,71,72,73,74,75,76,77,78,79,80,81,82,83,84,85,86,87,88,89,90], including viral or bacterial septic shock [91] and the autoinflammatory syndrome with MAS [91,92,93]. To dam inflammation, IVIG should be given at a dosage not exceeding 0.4 g/kg/day for three to five consecutive days (maximum total dosage is 2 g/kg) and the patient should be well hydrated. Higher IVIG dosage and/or poorly hydrated patients may increase the blood hyperviscosity, favoring coagulation, self-Ig aggregation with a transient complement activation, and/or the risk of the onset of rare side effects, such as acute aseptic meningitis [94].

## 5. Passive Immunotherapy

The infusion of plasma serum obtained from PCR-negative, recovered patients, containing IgG anti-SARS-CoV-2 (hyperimmune IgG-containing plasma; HIgCP), could be an attractive approach in newly infected subjects, based on previous experiences that are related to other viral infections, namely SARS-CoV, H5N1 avian influenza, and H1N1 influenza [95,96,97,98]. The administration of HIgCP could be useful to treat or prevent ARDS that is induced by SARS-CoV-2 infection and to accelerate virus clearance [99,100,101]. Even so, the need for a blood group match between donor and recipient makes the use of HIgCP less suitable than IVIG for large scale therapy. Preliminary clinical experiences are promising [102], but ongoing clinical trials are assessing the true effectiveness of this therapeutic strategy (NCT04321421) (last update posted: 27 April 2020; last access date: 9 May).

Finally, the administration of fully human mAbs specific to the receptor-binding domain (RBD) of the virus (responsible for its entry in the cell) [103,104] might be a valid alternative to HIgCP in controlling SARS-CoV-2 infection, because mAbs can be produced in large amounts, reproducible manner, with the same specificity, and they do not require a pre-evaluation of ABO blood groups match [103,104]. Recently, a SARS-CoV2-RBD-specific human neutralizing mAbCR3022, isolated from a single-chain variable antibody fragment (scFv) phage display library, which was constructed with lymphocyte RNA from convalescent SARS patient [105] has been reputed a promising therapeutic candidate for the treatment of COVID-19 infection [106]. Another mAb with potential therapeutic utility is the humanized mAb47D11 generated through hybridoma technology from splenocytes of H2L2 human Ig transgenic mice immunized with the spike protein. This mAb is capable of neutralizing both SARS-CoV and SARS-CoV-2 “in vitro”, by the binding to a conserved epitope on RDB, but the results are still preliminary (https://www.biorxiv.org/content/10.1101/2020.03.11.987958v1). 

## 6. Active Immunotherapy Approaches

Multiple attempts are being made to develop an effective vaccine against SARS-Cov-2. To date, there are 78 confirmed active projects, 53 of which are in exploratory development, 18 are at a preclinical stage, and five are being tested in phase I clinical trials [107]. Most of them target the surface-exposed spike protein in order to induce neutralizing antibodies.

The current vaccine platforms employ live attenuated viruses, inactivated viruses, non-replicating or replicating viral vectors, recombinant proteins, virus-like particles involved in DNA replication, and nucleic acid (DNA or mRNA) [108].

The only vaccine platforms that have been moved into phase I clinical trials include (1) the mRNA-based vaccine, from Moderna (NCT04283461) (last update posted: 4 May 2020; last access date: 9 May); (2) the DNA plasmid encoding the spike protein delivered by electroporation, from Inovio Pharmaceuticals (NCT04336410) (last update posted: 24 April 2020; last access date: 9 May); (3) the Adenovirus type 5 (Ad5) expressing the spike protein, from CanSino Biologicals (NCT04324606) (last update posted: 8 May 2020; last access date: 9 May); (4) the lentiviral vector system expressing viral proteins and immune modulatory genes to modify dendritic cells and activate T cells, from the Shenzhen Geno-Immune Medical Institute (NCT04276896) (last update posted: 19 March 2020; last access date: 9 May); (5) the lentiviral vector system expressing viral proteins and immune modulatory genes to modify artificial antigen presenting cells and to activate Tcells, also from the Shenzhen Geno-Immune Medical Institute (NCT04299724) (last update posted: 9 March 2020; last access date: 9 May) [107]. However, anti-SARS-CoV-2 vaccines need to be available very soon, and in large amounts, because the COVID-19 pandemic is still ongoing.

Among the vaccines in a clinical phase as candidates for SARS-CoV-2, them RNA-based vaccine seems to be more promising than other more traditional ones, because it is amenable to low-budget production of large quantities of vaccine. Although safe in pilot clinical trials, the antigen-specific immune response that is induced by RNA-based vaccines is lower than that observed in animal models [109].

Like RNA-based vaccines, DNA vaccines are easy to produce, cost-effective, and present a good safety profile and long term persistence of the immunogen. Even so, they have not been approved for human use due to their inability to evoke a strong enough immune response to be protective. Instead, viral vector-based vaccines, which are highly immunogenic, have been shown to generate efficient humoral and cell-mediated immune responses. However, the use of an adenovirus as a vector to carry the gene encoding the target protein, as in the case of Ad5 expressing spike, raises some concerns that are related to pre-existing immunity against adenovirus in the human population [110]. Lastly, the use of viral protein-derived lentiviral vectors raises some safety concerns that are related to the potential risk of mutagenesis [111].

It is not possible to predict which vaccine will be more effective in preventing SARS-CoV-2 infection. Only results from phase II/III trials will be able to suggest the more effective vaccine formulation. In this context, considerable attention should be paid to the remote possibility of a vaccine-triggered disease activation, as previously observed with animal models of anti-CoV vaccines [112].

## 7. Conclusions

Many antiviral and immunomodulant drugs are currently available for COVID-19, to be used alone or in combination, either at the initial disease stage or to protect patients from an inflammatory syndrome, preventing ARDS. However, most of these drugs are not virus-specific, with the exception of the hyperimmune Ig-containing sera obtained from individuals who recovered from the infection, but the effectiveness of this is still under study. The previous outbreaks, like the 2002 SARS and 2012 MERS experiences, have generated high expectations for a rapidly available vaccine. Unfortunately, and unexpectedly, all of the ongoing vaccine programs are still in clinical phase I, and most of them are employing platforms that have never been used before rather than the well-known platforms employed for the vaccines that are already commercially available, which have as of yet proven ineffective.

## Figures and Tables

**Table 1:** List of diseases for which treatment with human normal immunoglobulin for intravenous administration (IVIG) has been approved by the Food and Drug Administration (FDA) and/or European Medicines Agency (EMA).

Disease Denomination	IVIG Use Approved by		Rationale and/or Mechanism of Action	References
	EMA ^a)^	FDA ^b)^		
Primary immunodeficiencies (PID)	Yes	Yes	IgG replacement	[56]
Clinically manifest secondary immunodeficiencies (HIV, CLL, B cell depletion)	Yes	Yes	IgG replacement	[57,58]
Idiopathic thrombocytopenic purpura (ITP)	Yes	Yes	Fc receptor saturation	[59]
Kawasaki disease	Yes	Yes	Anti-inflammatory, binding to virus or superantigens	[60]
Chronic Inflammatory demyelinating polyneuropathy (CIDP)	Yes	Yes	Anti-inflammatory	[61]
Multifocal motor neuropathy	Yes	Yes	Not defined	[62]
Guillain-Barré Syndrome (GBS)	Yes	-	Anti-inflammatory	[63]

^a)^ Diseases for which treatment with IVIG has been approved by the EMA are continually being updated, as reported at “https://www.ema.europa.eu/en/ (last access date: 28 June 2020). ^b)^ Diseases for which treatment with IVIG has been approved by the FDA are continually being updated, as reported at “https://www.fda.gov/ (last access date: 3 May 2020).

**Table 2:** Diseases considered for the off-label use of intravenous human normal immunoglobulin (IVIG).

Disease	Rationale and/or Mechanism of Action	References
Prophylaxis in hematopoietic stem cell transplantation	Ig replacement	[64]
Infection disease conditions (toxemia, parvovirus 19)	Neutralization of pathogenic exogenous antigen, anti-inflammatory effects	[65,66,67]
Infections in solid organ transplantation, surgery, trauma, burns	Ig replacement	[68]
Idiopathic arthritis (especially the juvenile inflammatory form)	Fc-mediated	[69]
Systemic lupus erythematosus and lupus nephritis	Fc- and Fab- mediated	[70,71,72]
Autoimmune cytopenia (etc. autoimmune hemolytic anemia, Immune-mediated neutropenia)	Fc-mediated saturation of FcγRs, ADCC and CDC inhibition	[73]
Dermatomyositis and polymyositis	Fc-mediated	[74,75]
Catastrophic antiphospholipid syndrome	Fc- and Fab-mediated	[76]
Systemic capillary leak-like syndrome	Fc- and Fab-mediated	[30,77]
Vasculitides (ANCA associated)	Fc- and Fab-mediated	[78,79]
Skin autoimmune diseases (pemphigo, epidermolysis bullosa, atopic dermatitis, chronic urticaria)	mostly Fc-mediated, anti-inflammatory,	[80,81,82,83,84]
Myasthenia gravis	Fab-mediated	[85]
Small-fiber polyneuropathy	Not defined	[86]
Epilepsy	Not defined	[87]
Acquired factor VIII inhibitors	Fc- and Fab-mediated	[88]
Asthma	Anti-inflammatory	[89]
Recurrent pregnancy loss	Fc- and Fab- mediated	[90]

ADCC, antibody-dependent cell-mediated cytotoxicity; CDC, complement-dependent cytotoxicity.

## Abbreviations

APS	antiphospholipid antibody syndrome
ARDS	acute respiratory distress syndrome
CAPs	catastrophic anti-phospholipid syndrome
CLLS	capillary leak-like syndrome
CoV	coronavirus
COVID-19	coronavirus disease-2019
HIgCP	Hyperimmune IgG-containing plasma
RBD	receptor-binding domain
SARS	severe acute respiratory syndrome
WHO	World Health Organization
